# Skin manifestations associated with systemic diseases – Part II^☆^^☆☆^

**DOI:** 10.1016/j.abd.2021.06.003

**Published:** 2021-09-17

**Authors:** Juliana Martins Leal, Gabriela Higino de Souza, Paula Figueiredo de Marsillac, Alexandre Carlos Gripp

**Affiliations:** Service of Dermatology, Hospital Universitário Pedro Ernesto, Rio de Janeiro, RJ, Brazil

**Keywords:** Cardiovascular diseases, COVID-19, Diabetes mellitus, Endocrine diseases, Gastrointestinal diseases, Kidney disease, Pruritus

## Abstract

The skin, by reflecting internal processes, externalizes what happens inside the body in many diseases. Thus, the skin, as an organ, extrapolates its functions of protection, barrier and signals the existence of systemic diseases, expanding the importance of the dermatologist beyond the skin surface. Thus, the dermatologist investigates diagnostic hypotheses for conditions related to all systems and refers patients to the appropriate specialty. Combined with examination by a trained eye, the skin, due to its easy access, is still the ideal place for performing biopsies, which often clarify the diagnosis. This manuscript is the second part of the article on cutaneous manifestations of systemic diseases. In the first part, the cutaneous manifestations of the main rheumatologic and granulomatous diseases were described, and vascular manifestations were also addressed. In the present article, it will be discussed how metabolic, cardiovascular, kidney, and gastrointestinal diseases can manifest themselves in the integumentary system. Malignant diseases and their cutaneous implications, will also be discussed. Pruritus and its clinical cutaneous correspondence will be discussed. Finally, an update on cutaneous signs of SARS-CoV2 coronavirus infection will be presented.

## Introduction

The skin communicates with the body, often showing the first signs and/or symptoms of an internal disease, which may be the only expressions of systemic disorders.

In this second part of the article, the cutaneous manifestations of metabolic, cardiovascular, kidney, gastrointestinal and oncological diseases will be comprehensively addressed. In some of these diseases, it is possible to separate the cutaneous manifestations into specific ones, that is, in which histopathology confirms the diagnosis of the investigated disease and non specific ones when the cutaneous lesion does not have the histopathological characteristics of the systemic disease. This classification is not applicable to all diseases but will be applied whenever possible. Pruritus will also be discussed. Finally, a current review of the cutaneous manifestations of the new SARS-CoV-2 coronavirus infection will be presented, given its relevance in the current pandemic context.

## Metabolic diseases

### Diabetes mellitus

**Figure 1:**
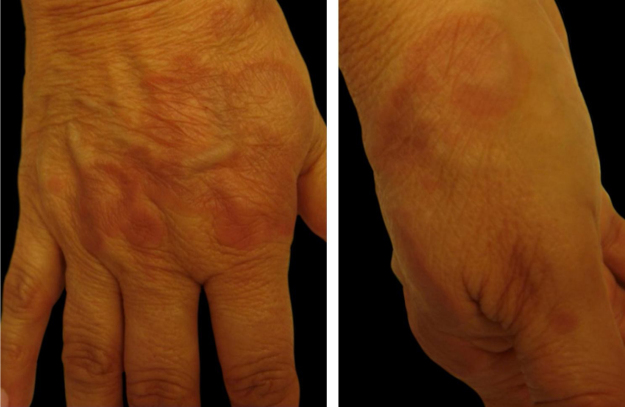
Granuloma annulare. Dr. Alexandre Gripp’s personal collection.
5 Acanthosis nigricans: it is characterized by intertriginous hyperpigmented plaques. The cervical and axillary regions are commonly affected in diabetic patients. It usually precedes the diagnosis of diabetes and may appear in a state of hyperinsulinemia with normal values ​​of glycosylated hemoglobin. It can also be seen in polycystic ovary syndrome and in obese individuals. It can manifest as a paraneoplastic including gastrointestinal malignancies. Additionally, drug-induced cases have been reported.^1^
6 Vitiligo: it is an autoimmune disease which often occurs concomitantly with other autoimmune diseases, such as insulin-dependent diabetes mellitus and thyroid disease. Achromic lesions often affect the extremities, facial, and cervical regions, as well as the trunk, and tend to be symmetrical and easy to diagnose.^1^
7 Carotenoderma: Yellowish skin and nails can be attributed to hypercarotenemia in diabetics.^1^
8 Lichen planus: About a quarter of patients with lichen planus have diabetes. Clinical examination shows characteristic lesions accompanied by localized pruritus on the ankles and wrists, with optional involvement of the mucosa. Other systemic diseases may also be associated with lichen planus, such as liver and bowel diseases, and thymoma.^1^
9 Acquired reactive perforating collagenosis: This is a rare skin disease seen in diabetic patients, as well as in chronic kidney disease and hyperuricemia. Its pathogenesis remains unknown. Although rare, the clinical picture is typical: erythematous pruritic umbilicated papules and nodules that represent transepidermal elimination of dermal debris. Koebner’s phenomenon is common in these patients (Fig. 2).^1^

1 Necrobiosis lipoidica: it is a chronic, non pruritic, granulomatous inflammatory disease with collagen degeneration. It usually starts with erythematous papules and slowly progresses to a yellowish-brown plaque with an atrophic core and telangiectasias. There is a higher prevalence in women than in men, and it is commonly located in the anterior region of the lower limbs and shows a symmetrical location. In 35% of cases, the lesions can ulcerate, and are commonly complicated by secondary bacterial infections. Most cases have a chronic course, but spontaneous improvement can sometimes occur.^1^
2 Scleredema adultorum of Buschke: it is characterized by slowly progressive, extensive induration, similar to scleroderma of the skin of the back, posterior cervical region, shoulders, and face. It is defined as the deposition of collagen and mucopolysaccharides in the dermis, with skin thickening, rigidity, and impaired motility, especially of the shoulders. It is a rare disorder, which may be underrecognized. It can also be found in other diseases such as neoplasms, paraproteinemia and infections. Huntley’s papules (small clustered erythematous papules) may be present. In extensive disease, the entire skin, as well as internal organs such as the lung, may be involved.^1^
3 Bullosis diabeticorum: usually in patients with long-term diabetes (it can also be an initial manifestation of the disease), which manifests as painless tense blisters, containing fluid, without signs of skin inflammation. They appear predominantly on the lower extremities, especially on the feet; however, they can affect the hands and trunk. The lesions grow quickly and heal within a few weeks. Approximately 0.5% of individuals with diabetes develop specific blisters over the course of the disease.^1^
4 Granuloma annulare (GA): it is characterized by normochromic to erythematous papules, forming annular-shaped plaques on the dorsum of the feet and hands or on the extensor surface of joints (such as the elbow). It usually appears in diabetic individuals, but the association with infectious diseases, such as hepatitis and HIV, and tumors, such as lymphoma and carcinoma, has also been described. The disease is often asymptomatic and lesions regress in the center of the plaque with hypo or hyperpigmentation. Most lesions heal spontaneously. The disseminated forms of GA are cosmetically unsightly and can be accompanied by some pruritus^1^ (Fig. 1).

**Figure 2:**
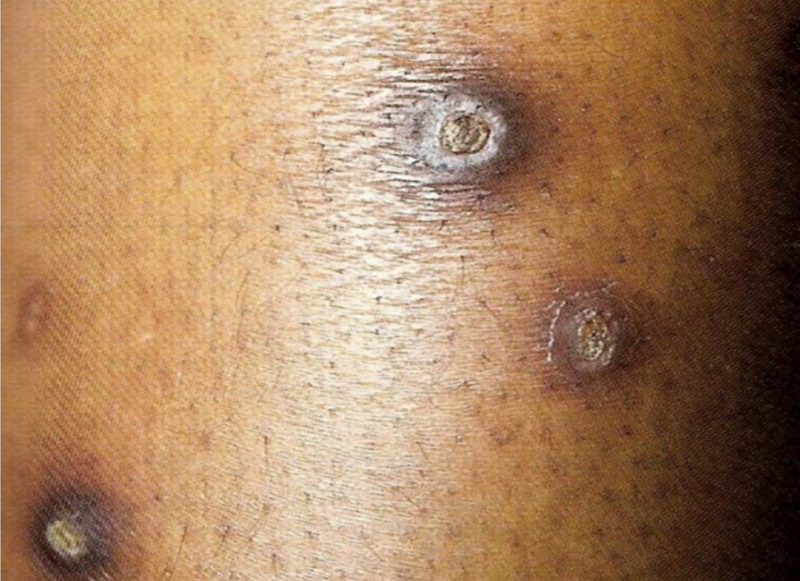
Acquired perforating dermatosis (Kyrle’s disease). Dr. Alexandre Gripp’s personal collection.
10 Diabetic angiopathy and neuropathy-associated skin diseases:
  a) Diabetic foot syndrome: the combination of diabetic angiopathy, neuropathy, and mechanical trauma plays an important role in its pathogenesis, which courses with a chronic ulcer, in which a quarter of patients develop complications such as infections and osteomyelitis.^1^
  b) Diabetic hand syndrome: diabetes can also cause musculoskeletal disorders on the hands, such as joint mobility limitation (JML), Dupuytren's disease (DD) (Fig. 3) and carpal tunnel syndrome (CTS). JML is characterized by thickening and rigidity of the periarticular connective tissue of the small joints of the hand, causing finger extension limitation. DD is caused by fibrosis and consequent thickening and shortening of the palmar fascia, resulting in flexion contractures. On the other hand, CTS is characterized by hypo- or dysesthesias in the innervation area of ​the median nerve of the hand.^1^

**Figure 3:**
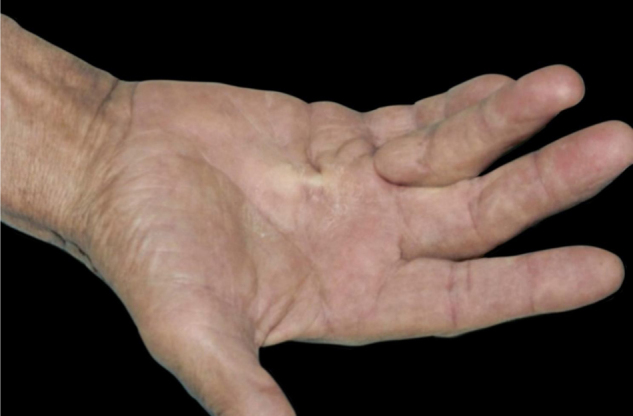
Dupuytren’s contracture. Dr. Alexandre Gripp’s personal collection.
  c) Diabetic dermopathy: it can be caused by minor trauma. It is a frequent manifestation in diabetic individuals and accompanies other diseases such as nephropathy, retinopathy or neuropathy. The disease tends to appear in the lower extremities of older men. The clinical finding are asymptomatic erythematous macules or fast-growing papules. The lesions have a recurrent course and spontaneously improve, resulting in brownish hypertrophic scars.^1^
11 Skin infections common in patients with diabetes: skin manifestations of bacterial and fungal infections are frequent in diabetics. Recurrent skin infections should be a reason for investigating diabetes mellitus.

*Staphylococcus aureus* can cause pyoderma (folliculitis, abscesses and impetigo). Streptococci are associated with ecthyma, cellulitis and contagious streptococcal impetigo (in this case, *Streptococcus haemolyticus*). Necrotizing fasciitis is a rare complication of *Streptococcus* infection; however, *S. aureus* or anaerobic bacteria may be involved. Fournier’s gangrene is a severe perineal or genitoanal variant of necrotizing fasciitis with a high mortality rate.

The skin of the otorhinolaryngological tract is also prone to bacterial infections in diabetics. Malignant external otitis is an invasive bacterial infection, commonly caused by *Pseudomonas aeruginosa,* and can have a lethal course. The infection usually starts asymptomatic and causes otalgia with purulent discharge. Severe cases can be extensive, reach the skull and the central nervous system, manifesting as meningitis and cerebritis.

Candidiasis is a common fungal infection in diabetics. *Candida albicans* is the most prevalent pathogen and can affect the skin and mucous membranes, as well as the periungual region (paronychia). Infection caused by dermatophytes (cutaneous dermatophytosis or onychomycosis) is also common.

Rhinocerebral mucormycosis is a serious disease, with high mortality, and is associated with diabetic ketoacidosis. It is mainly caused by species of the genus *Rhizopus*, *Rhizomucor*, and *Lichtheimia* (formerly called *Absidia*), opportunistic and angioinvasive fungi. Clinically, it starts as sinusitis with a serosanguinous nasal secretion that progresses to periorbital edema, erythematous-violet coloration, with signs of necrosis of the skin, nasal and oral mucosa. There are signs of toxemia, local pain and fever. There may be peripheral facial paralysis. The evolution is rapid for severe impairment of the central nervous system.

12 Complications of antidiabetic therapy
  a) Skin reactions caused by insulin: systemic allergic reactions include angioedema, generalized urticaria or anaphylaxis, flush, palmar-plantar or disseminated pruritus. Local reactions are urticarial or eczema like, with erythema, papules, and vesicles at the injection site, accompanied by pruritus. Serum sickness-like reactions may occur. Lipodystrophy, less common than allergic reactions, occurs after 6 to 24 months of continuous application of insulin doses always in the same location. The introduction of purified insulins has reduced the incidence of lipoatrophy.^1^
  b) Skin reactions induced by oral antidiabetics: macular rash, urticaria and erythema multiforme are observed. Photosensitivity can occur with tolbutamide and chlorpropamide. Lichenoid eruptions and rosacea-like lesions may occur in 1% to 5% of individuals using oral hypoglycemic agents. Of all oral hypoglycemic medications, sulfonylureas are the ones that most often cause reactions (second-generation drugs cause fewer cutaneous adverse effects than first-generation ones).
13 Pruritus: It can be associated with diabetes and will be discussed later.

### Thyroid diseases

1 Hyperthyroidism (associated skin conditions are shown in Table 1).

**Table 1:** Skin manifestations of hyperthyroidism.

Warm, moist and smooth skin
Blush
Palmar erythema
Hyperhidrosis
Diffuse thinning of the scalp
Onycholysis
Pretibial myxedema
Thyroid acropathy
Generalized pruritus
Chronic urticaria

Adapted from: Lause M et al., 2017.^2^
2 Hypothyroidism (associated skin conditions are shown in Table 2).

**Table 2:** Skin manifestations of hypothyroidism.

Dry skin
Cold and blotchy skin
Carotenemia
Myxedema
Macroglossia
Loss of the lateral third of the eyebrows
Rough and brittle hair

Adapted from: Lause M et al., 2017.^2^

Several autoimmune diseases may be associated with hypothyroidism, with the following having been reported: dermatitis herpetiformis, alopecia areata, vitiligo, and autoimmune urticaria.^2^

### Dyslipidemia

**Figure 4:**
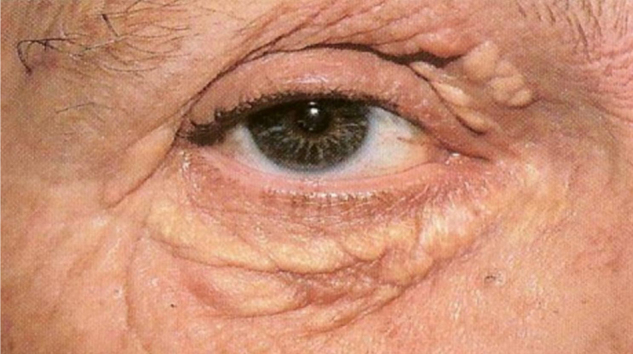
Xanthelasma. Dr. Alexandre Gripp’s personal collection.

1 Xanthomas
  a) Tuberous xanthoma: it appears as small, soft, yellowish, erythematous papules located on pressure areas (elbows, knees, gluteal region); they are painless and grouped together to form tumoral lesions. The presence of tuberous xanthoma suggests an elevation in serum cholesterol and LDL, but it can also be seen with an increase in triglycerides. There is a greater association with type III and type IIa mixed hyperlipidemia, a higher incidence of atherosclerotic vascular disease, and is rarely associated with secondary hypercholesterolemia.^3^
  b) Tendinous xanthoma: corresponds to firm nodules of varying sizes and are seen in areas over tendons (usually extensor tendons of the hands, knees, elbows, and Achilles tendon). They are associated with familial or secondary hypercholesterolemia, as well as cerebrotendinous xanthomatosis and beta-sitosterolemia. Patients with tendinous xanthomas have an extremely high incidence of atherosclerotic vascular disease.^3^
  c) Eruptive xanthoma: this is an abrupt, eruptive manifestation and is characterized by multiple papules measuring 1 to 4 mm, yellowish, with an erythematous halo. The lesions can coalesce to form tumor-nodular lesions. It most commonly occurs on pressure points and extensor surfaces of the upper limbs. More rarely, they may be diffusely spread over the trunk or mucous membranes. Unlike other xanthomas, eruptive xanthoma can be pruritic. They are associated with secondary hypertriglyceridemia and, more rarely, with the primary type (type IV, V and type I). Patients with eruptive xanthoma are at high risk for developing pancreatitis.^3^
  d) Xanthoma planum: it is the most frequent clinical manifestation and there are three types: xanthelasma, striated xanthoma and xanthoma planum. Xanthelasmae are polygonal, yellowish papules located on the eyelids (most commonly in the medial corner). At least 50% of patients with xanthelasmae do not have dyslipidemia. When serum lipids are abnormal (younger patients), cholesterol is usually the elevated fraction. Patients may also have corneal arcus as a sign of hypercholesterolemia. Some secondary hyperlipoproteinemias, such as cholestasis, can also be associated with xanthelasmae (Fig. 4).

Palmar striated xanthoma presents as flat, yellowish to orange lesions on the palmar folds, which only occur in patients with high levels of cholesterol and triglycerides.

Diffuse xanthoma planum usually covers large areas of the face, neck, chest, and upper limbs in individuals with or without dyslipidemia (particularly hypertriglyceridemia), but who often present with paraproteinemia, including those related to multiple myeloma.

### Adrenal

1 Cushing’s syndrome: corresponds to the set of clinical features mainly caused by hypercortisolemia. Cutaneous manifestations include “moon face”, buffalo hump, corresponding to deposition of adipose tissue in the dorsocervical region, the cervical region increased in diameter and shortenned due to the accumulation of supraclavicular fat, exophthalmia, due to the deposition of retroorbital fat, spontaneous hematomas, delayed wound healing, skin atrophy, striae distensae, hyperpigmentation, acanthosis nigricans, in addition to acneiform lesions (corticoid acne).
2 Addison’s disease/primary adrenal insufficiency: it is most commonly caused by autoimmune mechanisms. The main mucocutaneous manifestations are diffuse cutaneous, capillary, and mucosal hyperpigmentation. One can also observe the loss of axillary and pubic hair, in addition to hyperpigmented longitudinal ungual bands.
3 Pheochromocytoma: the only significant cutaneous manifestation of a pheochromocytoma is a very prominent paroxysmal flushing on the face, chest, and upper extremities. When pheochromocytomas occur as part of a genetic syndrome, the cutaneous manifestations of that syndrome may also be apparent.

### Hyperpituitarism

Acromegaly (associated cutaneous conditions are shown in Table 3).

**Table 3:** Mucocutaneous manifestations of acromegaly.

Macroglossia
Macrocheilia
Gingival hyperplasia
Coarse facial features
Cutis verticis gyrata
Hyperpigmentation
Acanthosis nigricans
Hyperhidrosis
Hypertrichosis
Nail changes

Adapted from: Lause M et al., 2017.^2^

### Hypopituitarism

Skin lesions may be the first indication for diagnostic suspicion. The skin and subcutaneous tissues become thin, the hair becomes sparse and the skin pale or yellowish. The signs of hypothyroidism may be evident and there may be symptoms of gonadotropin deficiency, especially reduced libido. Endocrine manifestations of hypopituitarism vary with the type, age of development and degree of hormone deficiency.

## Cardiovascular diseases

Unlike other organs, such as the liver (and the gastrointestinal tract itself), and the endocrine system, the cardiovascular system does not have any disease of its own that can have specific manifestations in the skin. There are several diseases that develop non specific cardiac and cutaneous manifestations, which can be classified as genodermatoses with cutaneous and cardiovascular involvement and cutaneous signs of systemic inflammatory or neoplastic diseases with cardiovascular manifestations. The following are examples:

### Genodermatoses

1 Carney complex: These are familial, dominant autosomal multiple endocrine neoplasias. There is skin and mucosal pigmentation (lentigines on the face, lips, eyelids, conjunctiva and oral mucosa), cardiac and cutaneous (and also breast) myxomas, and endocrine tumors, which include: growth hormone and prolactin-producing pituitary adenomas and primary pigmented adrenocortical nodular disease, testicular neoplasia, thyroid adenoma or carcinoma, and ovarian cysts.^4^ Its subtypes are the acronyms: LAMB and NAME. LAMB refers to lentigines, atrial myxomas and blue nevus. NAME stands for: nevi, atrial myxoma, myxoid neurofibroma, and ephelides.
2 LEOPARD syndrome: an acronym for lentigines, ECG conduction abnormalities, ocular hypertelorism, pulmonary stenosis, abnormal genitalia, retardation of growth, and sensorineural deafness.^5^
3 Ehlers-Danlos syndrome: characterized by several defects in the structure of collagen V, leading to changes on the skin (muscle-like pseudotumors, spontaneous hematomas), joints, and blood vessel walls. In 2017, the disease was classified into 13 variants. The disease can have a clinical manifestation spectrum, in which asymptomatic disease, expressed as non syndromic joint hyperflexibility, is the most frequent phenotype.^6^
4 Elastic pseudoxanthoma: genodermatosis of autosomal recessive inheritance due to mutations in the ABCC6 gene on chromosome 16, characterized by the mineralization (deposit of calcium and other minerals) and elastic fiber degeneration, which lead to skin (yellowish pruritic plaques on the cervical and axillary regions and other flexures), vascular (narrowing, occlusion and aneurysms of peripheral or cerebral arteries – with intermittent claudication of the lower limbs, cardiac angina or cerebral ischemia/hemorrhage and gastrointestinal hemorrhage) and ophthalmologic involvement, expressed by retinal angioid streaks, with reduced visual acuity.^7^
5 Hereditary hemochromatosis: an autosomal recessive disease that causes iron deposition in hepatocytes, myocardial muscle fibers, and other cells. The main clinical cutaneous manifestations are brownish to grayish hyperpigmentation of the skin, rapid tanning with minimal exposure to the sun (caused by melanin, which follows hemosiderin deposits in photoexposed areas of the body – mainly on the head, and also on the genitalia, nipples, scars, and flexures). Atrophy and ichthyosiform xerosis, hair loss and koilonychia, hyperpigmentation of the oral mucosa, reduction of tooth enamel and saliva are also observed. The practice of phlebotomy does not rapidly revert skin pigmentation.
6 Tuberous sclerosis: it is caused by loss-of-function mutations of the TSC1 or TSC2 tumor suppressor genes. In 2/3 of the cases, it is a de novo mutation and in 1/3 it is hereditary. On the skin, there are facial angiofibromas (in 75% of individuals), periungual (20%–80% of cases) and oral fibromas (50% of cases), fibrotic plaques in the frontal region (25% of cases), hypochromic macules (90 % of cases) and “shagreen” plaques, which are connective tissue nevi present in 50% of patients. It is currently believed that facial angiofibromas are induced by ultraviolet radiation, and it is possible that photoprotection reduces their appearance.^8^
7 Fabry disease: this is an inborn error of metabolism, in which there is no lipid catabolization. Dermatological manifestations begin in childhood, with acroparesthesia and intolerance to heat, accompanied by nausea, vomiting, abdominal and neuropathic pain. During the lifetime, angiokeratomas and telangiectasias appear in the gluteal region and thighs.^9^
8 Systemic amyloidosis: It can be classified as hereditary (ATTR, ALys, Ab2M, AFib, AApoA1, AGel types) or acquired (AL, AA, ATTR, ALect1, Ab2M types). AL amyloidosis is the most frequent form (associated with plasma cell dyscrasias, most commonly multiple myeloma) and the one with the most frequent cutaneous manifestations (but in only 1/3 of cases). Macroglossia and periorbital purpura may be present. Note that isolated periorbital purpura occurs in other forms of amyloidosis. In addition to these clinical manifestations, other findings that indicate the need for investigation of amyloidosis are onychodystrophy with thin nail plates, onychorrhexis, distal pterygium, and onychoschizia.^10^ The exclusively cutaneous forms of amyloidosis caused by cytokeratin 5 – CK5 deposition (macular amyloidosis and lichen amyloidosus) do not progress to systemic disease.

### Inflammatory and neoplastic diseases

1 Rheumatic fever: this is an autoimmune disease, triggered by antibodies against group A beta-hemolytic Streptococcus, which in the acute phase, in addition to fever, causes cutaneous manifestations such as erythema marginatum, i.e., plaques with non-pruritic erythematous circinate borders and a clear center, in addition to migratory joint impairment.
2 Carcinoid syndrome: it manifests as abdominal pain, diarrhea, flushing (on the face, neck and chest), telangiectasias, and bronchospasm due to the release of several substances by metastatic or extra-hepatic neuroendocrine tumors (from enterochromaffin or Kulchitsky cells). There may be symptoms of niacin deficiency (pellagra) due to the use of tryptophan (the precursor of niacin), which is the raw material for the production of the secreted hormones.^11^

### Thromboembolic phenomena

Thromboembolic phenomena of different origins have cutaneous manifestations that are the “clues” suggesting these diagnostic hypotheses. Lesions usually present as petechiae, retiform purpura, and splinter hemorrhages. The possible causes are antiphospholipid antibody syndrome and other coagulopathies. Cholesterol emboli may result in livedo reticularis, retiform purpura, and eosinophilia. Leukocytoclastic vasculitis can occur in the context of infectious diseases such as hepatitis, bacterial or fungal infective endocarditis, and in certain cases is associated with p-ANCA positivity. Infectious endocarditis can occur with Janeway lesions that correspond to erythematous to purpuric macules on the palms and soles, usually at the base of the first and fifth fingers, and to Osler’s nodes, which occur as painful erythematous-violet papules or nodules on the same locations of Janeway lesions.

### Other isolated skin manifestations as possible semiological observations of heart disease

1 Digital clubbing: it suggests heart disease and/or chronic lung disease, or even lung cancer.
2 Cyanosis: cyanotic heart disease, especially in children with congenital heart disease; methemoglobinemia (this, in addition to cyanosis, may be accompanied by grayish skin), intense vasoconstriction (Raynaud’s syndrome).
3 Diagonal crease in the ear lobe (Frank's sign): possibly related to coronary artery disease and less scientific evidence associates it with carotid atherosclerosis.
4 Lower limbs edema: it can be caused by congestive heart failure or right ventricular failure.

## Renal diseases

The skin manifestations of kidney diseases can be classified into:

1 Skin lesions associated with uremia:
  a) Manifestations that have a specific histopathological diagnosis, but which is not specific for the kidney disease itself:

Calcinosis cutis: a late complication of renal failure, it occurs in 1% of individuals with chronic kidney disease (CKD) on hemodialysis (HD). It is a metastatic type of disease, caused by an increase in the calcium/phosphorus ratio. Clinically, the lesions are asymptomatic or painful indurated papules, plaques or nodules that ulcerate and eliminate yellowish-white material. They can be seen on the X-ray as radiopaque deposits in the subcutaneous tissue and on histopathology as a bluish homogeneous material in the dermis, which can be confirmed by staining with the Von Kossa method.

**Figure 5:**
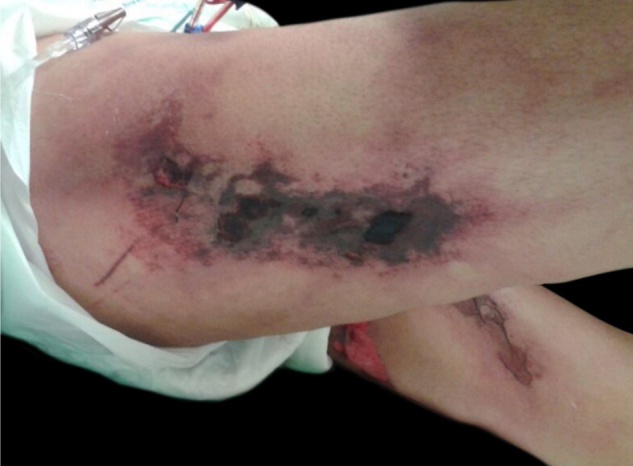
Calciphylaxis. Dr. Ana Luisa Sampaio’s personal collection.

Calciphylaxis: it also occurs in 1%–4% of CKD patients on HD with secondary hyperparathyroidism and increased calcium/phosphorus ratio (> 60 to 70 mg^2^/Dl^2^). This is a thrombotic vasculopathy due to calcium deposition in the middle layer of the skin arterioles, in addition to intima layer hyperplasia. The lesions are initially similar to livedo reticularis that progress to extremely painful and symmetrical retiform purpura plaques with ulcer formation and necrosis. It has high morbidity, usually due to sepsis caused by secondary infection of the necrotic areas. Lesions are commonly located in areas with a higher concentration of adipose tissue, such as the gluteal region, abdomen and lower limbs (thighs and legs). They are more frequent in women. It is believed that individuals with thrombophilia (changes in proteins C and S and presence of antiphospholipid antibodies) are more likely to develop calciphylaxis. Other factors include hypercalcemia, use of calcium-based phosphate binders, vitamin D replacement, hyperphosphatemia, hyperparathyroidism, diabetes mellitus, obesity, use of systemic corticosteroids, immunosuppression, and trauma^12^ (Fig. 5).

Acquired perforating dermatosis (Kyrle’s disease): associated with diabetes mellitus and CKD. It affects 4.5%–10% of individuals on HD. The lesions comprise intensely pruritic hyperchromic nodules or papules, with umbilication and hyperkeratosis at the top of the lesion, located on the extensor surfaces of the extremities, and have a differential diagnosis with prurigo nodularis. They can also occur on the scalp, trunk and gluteal region. They are histopathologically specific: there is acanthosis, cup-shaped keratotic plugs, elimination of tissue debris through the epidermis and perilesional inflammatory infiltrate consisting of neutrophils and lymphocytes. Other acquired perforating dermatoses, such as perforating folliculitis and reactive perforating collagenosis may also occur (Fig. 2).^13^

Systemic nephrogenic fibrosis: it is a rare manifestation related to gadolinium contrast (gadodiamide and gadopentetate) used in MRI scans and associated with genetic predisposition (HLA-A2) and an inflammatory state that allows the entry of gadolinium into tissues. The contrast is phagocytosed by macrophages in the tissues and triggers the fibrotic process. Clinically, symmetrical, indurated plaques are observed, ranging from erythematous to hyperchromic, initially pruritic, particularly on the extremities. Later, they present as skin atrophy, hair loss, and “*peau d’orange*” appearance. It can develop limb contractures and 5% of the cases show a fulminant evolution with multiple-organ involvement. Skin biopsy demonstrates thickened collagen bundles with clefts, mucin deposition, and proliferation of spindle cells that are positive for CD34 and type I procollagen, as well as multinucleated cells positive for CD68 and factor VIII. The differential diagnosis is with other diseases that present with fibrosis (scleromyxedema, systemic sclerosis, eosinophilia-myalgia syndrome, toxic oil syndrome, and eosinophilic fasciitis).^12^

**Figure 6:**
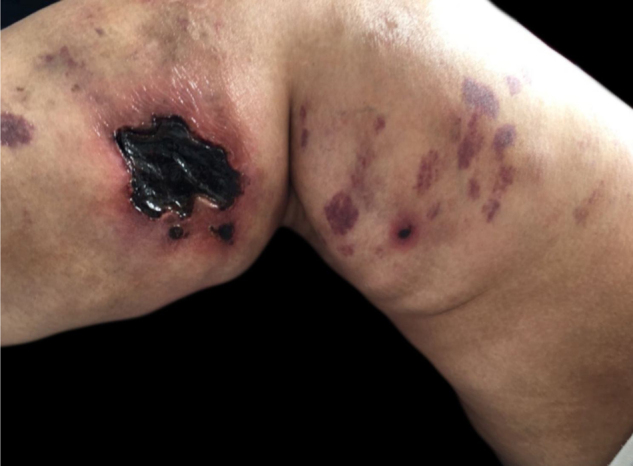
Intravascular oxalosis. Dr. Ana Luisa Sampaio’s personal collection.

Oxalosis: it is a rare disease and comprises two forms: primary and secondary oxalosis. Type I primary oxalosis occurs in 80% of cases of primary oxalosis and is caused by an autosomal recessive deficiency of the enzyme alanine-glyoxylate aminotransferase (GAT), and then there is no metabolization of oxalate into glycolate, which thus accumulates in tissues and causes CKD in most patients aged 20–30 years. Type II is caused by a deficiency of the enzyme complex glyoxylate reductase/hydroxypyruvate reductase. It is milder than type I, with a recurrent clinical picture of nephrolithiasis and rarely progressing to CKD. Type III is caused by a mutation in the HOGA1 gene, leading to loss of function of the mitochondrial enzyme 4-hydroxy-2-oxoglutarate aldolase and is clinically characterized by recurrent nephrolithiasis in childhood (in the first decade of life). Primary oxalosis is manifested by intravascular oxalate deposits in the kidneys (leading to end-stage kidney disease), myocardium, bones, and skin, where it clinically resembles calciphylaxis, with livedo reticularis and retiform purpura, progressing to ulceration and necrosis.^14^ The lesions tend to be more superficial and less painful than calciphylaxis lesions and to be accompanied by hardened plaques similar to those of nephrogenic fibrosis. (Fig. 6) Secondary oxalosis is caused by a large intake of oxalate or its precursors (spinach, beetroot, rhubarb, vitamin C, and ethylene glycol intoxication), or by increased absorption by the colon, pyridoxine deficiency, or CKF (due to lower renal excretion). It leads to extravascular oxalate deposits, which translate clinically into milia-like papules and nodules in the dermis.^15^ The histopathological diagnosis of both forms is identical and specific and shows calcium oxalate crystals (yellow-brownish, rectangular or triangular-shaped, needle-like structures arranged in rosettes, refringent under polarized light) in tissues or intravascular locations. Von Kossa staining may show irregular calcium deposits. There is a scarce inflammatory infiltrate and some foreign-body giant cells.

**Figure 7:**
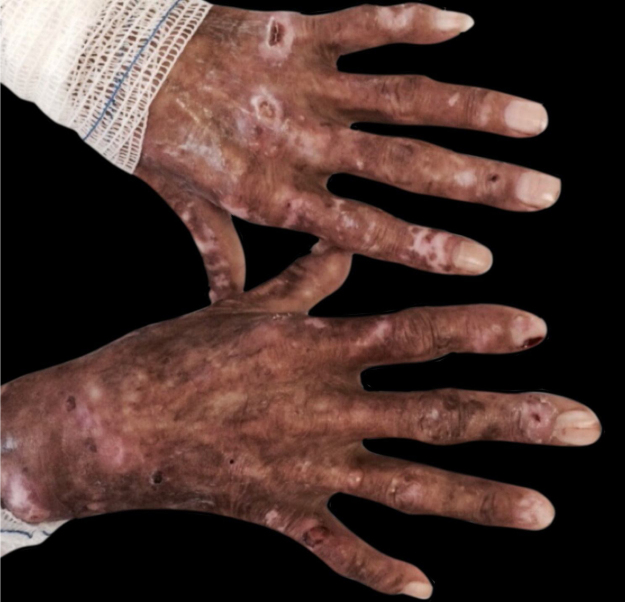
Porphyria cutanea tarda. Dr. Ana Luisa Sampaio’s personal collection.

Porphyria cutanea tarda: it occurs in 1%–18% of patients with CKD on HD due to insufficient excretion of uroporphyrins and their consequent accumulation in the skin, leading to photosensitivity. Porphyrins are not adequately dialyzed. Excessive serum iron and hepatitis C are factors that increase the predisposition to its occurrence. The patient has bullous vesicles and erosions in areas exposed to trauma such as hands, feet, forearms, and even the face, which regress with the formation of scars and milia. Hypertrichosis and hyperpigmentation of the face can occur. Histopathology shows a subepidermal cleft, festooning of the papillary dermis and scarce inflammatory infiltrate. Direct immunofluorescence shows deposits of IgG and C3 at the dermoepidermal junction and around the vessels. There is an increase in serum iron, ferritin, and uroporphyrin levels, an increase in isocoproporphyrins in the feces, and an increase in uroporphyrins I and III in the urine (Fig. 7).^13^

Pseudoporphyria: it is also observed in 1%–18% of cases. There is no increase in porphyrins. It can be triggered by drugs such as amiodarone, furosemide, tetracyclines, isotretinoin, naproxen, nalidixic acid, and exposure to ultraviolet light. The clinical picture is similar to that of porphyria cutanea tarda, but without hypertrichosis and hyperpigmentation of the face.^12^

b) Non specific alterations from the histopathological point of view:

Pruritus caused by kidney disease (50%–90% of cases).

Cutaneous changes secondary to pruritus: excoriations, prurigo nodularis, post-inflammatory hyper or hypopigmentation, chronic lichen simplex.

Xerosis: 50%–80% of patients on hemodialysis. The pathophysiology of skin surface dryness is not well understood and causes pruritus.

Dyschromia: due to the accumulation of non dialyzable substances, such as hemosiderin (grayish-brown pigmentation), carotenes, and urochromes (yellowish color), and melanocyte-stimulating hormone, revealed by brownish skin in a photoexposed area.^12^

Uremic frost: (1%–3% of cases) it occurs with high serum levels of urea (250 to 300 mg/dL), due to its elimination through sweat and its accumulation on the skin surface after evaporation of water in sweating areas.

c) Nail changes: half-and-half nails (Lindsay's nails) occur in 40% of individuals on hemodialysis and are caused by azotemia and regress after kidney transplantation; Muehrcke's lines (double white transversal lines associated with hypoalbuminemia – with values < 2.2 g/dL – in nephrotic syndrome); other alterations mentioned in the literature: changes on the nail fold capillaries; melanin pigmentation of the distal nail plate, leukonychia, koilonychia, and splinter hemorrhages.^12^
d) Hair alterations occur in 30% to 50% of cases: alopecia due to telogen effluvium, chronic disease or the underlying disease that led to CKD. Loss of shine and discoloration of hairs is also reported.
e) Mucosal changes: they occur in 50% to 90% of cases. Xerostomia and uremic breath occur. Macroglossia can occur in the context of CKD associated with systemic amyloidosis.
f Skin lesions as a consequence of kidney transplantation: due to the use of corticosteroids and their short and long-term consequences, cyclosporine and immunosuppressants.
g Skin lesions caused by the primary disease that led to CKD: cutaneous lesions associated with systemic lupus erythematosus, genetic diseases (Fabry disease, Birt-Hogg-Dubé syndrome, nail-patella syndrome, tuberous sclerosis, among others).

## Gastrointestinal diseases

1. Inflammatory Bowel Disease (IBD)

IBDs are chronic diseases that are closely related to the skin, resulting in many implications that contribute to increased morbidity.^16^

Oral ulcers may represent contiguity lesions of the intestinal disease, with similar histopathology. However, they can also be a reaction to IBD. Aphthous stomatitis is present in more than 7% of all patients. Multiple painful ulcers with erythematous borders, periodontitis, and peristomatitis may appear.^16^

Pyostomatitis vegetans is a rare eosinophilic disease that leads to the formation of friable pustules, ulcers, and yellowish vegetating plaques on the oral mucosa and other mucous membranes. When it affects the skin, it is called pyodermatitis-pyostomatitis vegetans.^16^, ^17^, ^18^

Erythema nodosum (EN) consists of painful erythematous-violaceous nodules, most typically found on the anterior surface of the tibial region, and it is present in up to 15% of patients with Crohn’s disease (CD) and 10% with ulcerative colitis (UC). Although up to 90% of EN cases are associated with bowel disease activity, its severity is not necessarily proportional. Systemic symptoms, such as fever and arthralgia, may be present.^16^, ^17^, ^18^

Pyoderma gangrenosum is a neutrophilic dermatosis that is often debilitating, which courses with dark red or violet, single or multiple painful ulcers with irregular, raised and undermined edges. It ranges in size from 2 to 20 cm and may be accompanied by fever, arthralgia and/or myalgia. It has a higher prevalence in women and individuals of African descent.^16^, ^17^, ^18^

Sweet’s syndrome, also classified as a neutrophilic dermatosis, is more rarely associated with IBD. It usually courses with fever, ocular alterations, leukocytosis, painful and asymmetrical erythematous papules, plaques, and nodules across the body.^16^, ^17^, ^18^

The pathogenesis of bowel-associated dermatosis–arthritis syndrome (BADAS) is based on excessive bacterial proliferation, causing the release of antigens and, as a consequence, the deposition of immune complexes. It is characterized by recurrent episodes of fever, abdominal pain, arthritis and may present with macules, papules, vesicles, and pustules with or without local pain.^17^

Vasculites are common. They usually present with palpable purpura, ulcers, nodules, digital vasculitis, and maculopapular exanthema. The most often described subtypes are leukocytoclastic, necrotizing and lymphocytic vasculitis. The most often associated necrotizing vasculitis is polyarteritis nodosa (PAN).

There are also manifestations of malabsorption secondary to gastrointestinal disease. This is the case of follicular hyperkeratosis or phrynoderma caused by vitamin A deficiency, angular cheilitis due to vitamin B12 deficiency, scorbutus due to vitamin C deficiency, and zinc enteropathic acrodermatitis, for instance.^17^, ^18^

Currently, with the advent of the use of immunobiologicals, alterations after the use of anti-TNF therapy may paradoxically appear, as psoriasiform and/or eczematous lesions, in addition to vasculites, lupus-like syndrome, among others.^16^

a) Crohn’s Disease

The cutaneous alterations of CD can be divided into specific and non specific. Specific alterations are caused by continuity such as oral ulcers, abscesses, fissures, and perianal or peristomal fistulas; or by metastatic Crohn’s disease (mCD), which can produce papules, erythematous infiltrated plaques, nodules or ulcers, distant from the original sites of disease involvement, with or without local pain.^16^, ^17^, ^18^, ^19^

With its poorly understood pathophysiology, mCD has a heterogeneous presentation and may appear before, during or after gastrointestinal symptoms occur. It affects both sexes equally and often affects the perineal region with edema, erythema, and fissures.^19^

Non specific manifestations may be related to skin reactivity due to the immunological mechanisms of CD; they may be independent diseases, but associated with CD or complications secondary to the lesions caused by CD (Table 4).^18^

**Table 4:** Non specific manifestations of Crohn’s Disease.

Skin reactivity
Aphthous stomatitis
Erythema nodosum
Pyoderma gangrenosum
Sweet’s Syndrome
Bowel-associated dermatosis–arthritis syndrome (BADAS)
Pyostomatitis vegetans
Leukocytoclastic vasculitis
Independent associated diseases
Psoriasis
Secondary amyloidosis
Vitiligo
Epidermolysis bullosa acquisita
Alopecia areata (AA)
SLE
Secondary complications
Acrodermatitis enteropathica
Angular cheilitis

Adapted from: Gravina et al., 2016.^18^

b) Ulcerative colitis

Between 5% to 11% of patients with UC may have skin alterations. Pyoderma gangrenosum and pyostomatitis vegetans are more frequent in UC than in CD. Leukocytoclastic vasculitis, erythema nodosum, and even more rarely, oral lesions have also been described.^16^, ^17^

2. Celiac disease

The most common extraintestinal manifestation is dermatitis herpetiformis (DH). It is caused by the deposition of immune complexes with IgA. DH is manifested by pruritic papules and vesicles that are preferentially located on the elbows, knees, and buttocks.^20^

3. Peutz-Jeghers syndrome

Peutz-Jeghers syndrome is a genetic disease characterized by intestinal hamartomatous polyposis and typical perioral hyperpigmentation.^21^ The pigmented lesions are lentigines, which may be present from birth and are preferentially located around the lips, oral mucosa, tongue, and nose.^22^

4. Cronkhite-Canada syndrome (CCS)

CCS is a rare disease the etiology of which is not well-established, characterized by adenomatous intestinal polyposis. On the skin, areas of lentiginous hypermelanosis, atrophic nail changes, and alopecia are typical. The alopecia may have characteristics suggestive of alopecia areata, and may also affect the eyebrows, eyelashes, axillary and pubic regions; in addition, after periods of disabsorption, it may be associated with acute telogen effluvium. Vitiligo, systemic lupus erythematosus (SLE), scleroderma, and hypothyroidism have been described in patients with CCS.^23^

5. Liver diseases

There are several diseases that affect the liver and biliary tract, as well as the mucous membranes and the skin. Cirrhosis, hemochromatosis, viral hepatitis, and also porphyria cutanea tarda and Wilson's disease will be addressed.

a) Cirrhosis

Liver cirrhosis can cause different semiological signs. Palmar erythema, vascular spiders, arteriovenous (facial) hemangioma, colateral circulation (medusa head pattern), flushing, plethoric facies, angioendotheliomatosis, pruritus, nail changes, hair loss, thinning of body hair, vasculitis, urticarial reactions, and jaundice are common manifestations.^24^

Nail changes are non specific and may appear in other diseases, such as nephrotic syndrome or after chemotherapy, for instance. Even so, they are classically associated with Terry’s nails, presenting a white nail bed with a pink or brownish distal band; and Muehrcke’s nails, with transversal white stripes.^24^ Digital clubbing and Dupuytren’s contractures can also be found.

b) Hemochromatosis

See the topic on cardiovascular diseases above.

Alterations secondary to liver cirrhosis may also be present.

c) Viral hepatitis

There are several cutaneous findings related to viral hepatitis. Jaundice and urticaria can be found in all of them. Urticarial vasculitis may be seen in hepatitis B and C.^24^

In patients with acute hepatitis B, periorbital edema, rash, lichenoid reactions, serum-like disease, and PAN may occur.^24^

Mixed cryoglobulinemia, vasculitis, exanthema, Gianotti-Crosti syndrome are described in the chronic picture.^24^, ^25^

Lichen planus, lichenoid lesions, granuloma annulare, PAN, and livedo reticularis have been described after immunization for hepatitis B.^25^

Hepatitis C is related to pruritus, cryoglobulinemia, porphyria cutanea tarda, vasculitis, livedo reticularis, lichen planus, Sjögren’s syndrome, urticaria, and PAN.^24^, ^26^

Less commonly associated are EN, erythema multiforme, nevoid telangiectasia, pyoderma gangrenosum, vitiligo, nail dystrophy, psoriasis, Behcet’s syndrome, granuloma annulare, and porokeratosis.^26^

Acral necrolytic erythema that presents with lichenified plaques and erythematous edges that may show desquamation, pruritus, and pain, is a rare disease and is associated with chronic hepatitis C.^24^

Hepatocarcinoma, one of the complications of chronic hepatitis C virus infection, has a well-established relationship with mixed cryoglobulinemia, porphyria cutanea tarda (PCT), and lichen planus.^24^

d) Porphyria cutanea tarda

Porphyria cutanea tarda (PCT) is a disease caused by a deficiency of the hepatic enzyme uroporphyrinogen decarboxylase, which alters the heme synthesis and affects the liver and the skin. Photosensitivity is the rule and it manifests as bullous vesicles that usually evolve with milia, pigmentary changes and scarring. Other findings are hypertrichosis and fibrous scarring.^24^

e) Wilson’s Disease

Wilson’s Disease (WD) is a disorder of copper metabolism that leads to its accumulation in the liver, but also in other organs and tissues. Mucocutaneous manifestations may occur as a result of this deposition, secondary to treatment with D-penicillamine or as a consequence of liver cirrhosis. The most described alterations are distal hyperpigmentation on the lower limbs, blue lunula, and Kayser–Fleischer rings (pigmentation circle in the periphery of the cornea).^27^

D-penicillamine, a drug used to treat Wilson's disease, can alter collagen biosynthesis. Thus, it can contribute to the appearance of anetoderma, elastosis perforans serpiginosa, cutis laxa, and pseudoxanthoma elasticum. After long-term use, it can also predispose to hypersensitivity reactions and bullous diseases.^27^

A greater number of subcutaneous lipomas has been observed in patients with WD; however, their pathophysiology remains to be elucidated.^27^ Freg et al., in 2016, described the case of a 17-year-old female patient who developed pyoderma gangrenosum.^28^

## Oncological diseases

Many skin conditions can be correlated with internal malignancies in specific or non specific ways. Some criteria proposed by Curth can assess whether the association between skin lesions and neoplasias is more or less likely, as explained in Table 5.^29^

**Table 5:** Curth’s postulates (criteria for the association between dermatosis and neoplasia).

Simultaneous onset
Parallel course
Neoplasm uniformity (site or cell type)
Statistical association
Genetic association

Adapted from: Curth HO et al, 1976.^29^

### Proliferative and inflammatory dermatoses

1 Acanthosis nigricans: its association with neoplasia should be suspected in case of sudden onset in a non obese individual in the absence of endocrinopathy. It is most commonly related to adenocarcinoma of the stomach, although it can be associated with other tumors in the abdominal cavity (gastrointestinal and genitourinary tracts). In more than 95% of cases, the tumors are adenocarcinomas.^30^
2 Bazex syndrome (acrokeratosis paraneoplastica): this is a psoriasiform eruption on acral surfaces that can progress to palmoplantar keratoderma and nail dystrophy.^30^ Ears, nose, the buccinator region, hands, feet, and knees are frequently affected.^31^ It is associated with squamous cell carcinomas of the upper aerodigestive tract (larynx, pharynx, trachea, bronchi, upper esophagus), usually accompanied by metastases to cervical lymph nodes.^30^ More rarely, it can occur in the context of other solid neoplasms and lymphomas, and investigation of the head, neck, and pelvis is indicated.
3 Bullous dermatoses: these are more likely to be related to neoplasms if the direct immunofluorescence of a skin biopsy is negative (or if there are linear deposits of IgA along the basement membrane) and if there are exuberant mucosal lesions.

Paraneoplastic pemphigus: It is associated with malignancies of the lymphoreticular system (Castleman’s disease, non-Hodgkin’s lymphoma, thymoma, follicular dendritic cell sarcoma, chronic lymphocytic leukemia).^32^ There may be multisystem involvement with bronchial lesions leading to respiratory failure and it has a poor prognosis.

Anti-epiligrin cicatricial pemphigoid secondary to adenocarcinomas.^33^

Dermatitis herpetiformis: increased relative risk of intestinal lymphoma, as seen in celiac disease itself.^34^

Epidermolysis bullosa acquisita: it is rarely seen in patients with lymphoreticular system tumors.

Porphyria cutanea tarda: related to liver tumors, but this association seems to be incidental, as hepatitis C virus infection is a risk factor for both.

4 Dermatomyositis: 10% to 30% of dermatomyositis patients may have associated neoplasms (overlapping syndromes and infantile dermatomyositis are rarely associated with neoplasms). When there is associated neoplasia, the disease is more resistant to treatment with corticosteroids, requiring the use of adjuvant immunosuppressants. Breast and ovarian cancer in women and lung and prostate cancer in men are strongly associated with dermatomyositis, and less so with polymyositis. The neoplasm is usually found within the first year of the disease.^35^
5 Eruptive seborrheic keratoses (Leser-Trelat sign): it can be understood as a variant of acanthosis nigricans and, therefore, it can also be related to adenocarcinomas of the gastrointestinal tract, although it has also been described as secondary to tumors of the female reproductive system and lymphoproliferative disorders.^36^
6 Erythroderma, generalized pruritus and ichthyosis: there is a predominant association with lymphoproliferative malignancies, as well as solid neoplasms. Screening for neoplasms is recommended in every patient with erythroderma of undefined etiology. Pityriasis rotunda seems to be a variant of ichthyosis acquisita and is correlated with hepatocarcinoma.
7 Figurate erythema: only erythema *gyratum repens* seems to be associated with malignancies.^37^ Lung cancer is the most common neoplasm, followed by breast, genitourinary, and gastrointestinal tumors.
8 Hypertrichosis lanuginosa acquisita: it is one of the most consistent cutaneous associations with neoplasms,^38^ with adenocarcinoma of the gastrointestinal tract being the most common.
9 Thrombophlebitis migrans (Trousseau’s syndrome): association with pancreatic, lung, stomach, prostate, and hematopoietic system neoplasms.^39^
10 Multicentric reticulohistiocytosis: nodular skin lesions with a predilection for the hands, associated with solid organ and lymphoreticular neoplasms.
11 Mycosis fungoides: it is primary cutaneous T-cell lymphoma and may be associated with Hodgkin’s disease and non-Hodgkin’s lymphoma.
12 Necrobiotic xanthogranuloma: association with multiple myeloma.
13 Paget’s disease, mammary and extramammary: possible association with metastatic adenocarcinoma of the breast, neoplasms of the gastrointestinal or genitourinary tract, depending on the area of ​​continuity of the skin lesions.
14 Neutrophilic dermatoses and pyoderma gangrenosum: related to myeloid leukemia, mainly in the bullous variant of pyoderma gangrenosum.
15 Pachydermoperiostosis: association with lung cancer.
16 Tripe palm: if isolated, it is associated with lung cancer. When seen in addition to acanthosis nigricans, an association with gastric cancer should be investigated.
17 Vitiligo: may be associated with melanoma, especially if it appears after the 4^th^ decade of life.

### Hormone Secretory Syndromes

1 Carcinoid syndrome: see the topic on cardiovascular diseases above.
2 Ectopic adrenocorticotropic syndrome: intense and more pronounced hyperpigmentation than is usually seen in Cushing’s syndrome associated with small-cell lung carcinoma.
3 Glucagonoma syndrome: it is associated with glucagon-producing pancreatic neoplasms and has necrolytic migratory erythema as its cutaneous manifestation.

Hereditary syndromes associated with internal neoplasms (Table 6).

**Table 6:** Inherited syndromes associated with internal neoplasms.

Syndromes	Cutaneous manifestation	Type of associated neoplasia
Birt-Hogg-Dubé	Acrochordons + benign follicular tumors in the head and neck	Kidney cancer
Cowden’s Disease	Perinasal and central-facial trichilemmomas: + Keratotic papules on the face, neck, ears and hands + Multiple papules on the oral mucosa + Lipomas + Hemangiomas	Breast cancer + +
		Thyroid adenocarcinoma + Squamous cell carcinoma of the skin
Gardner's syndrome	Epidermoid cysts + fibromas + lipomas + desmoid tumors	Malignant transformation of adenomatous intestinal polyps + CNS neoplasia
Hereditary leiomyomatosis and renal cell cancer	Cutaneous leiomyomas	Renal cell cancer
Multiple endocrine neoplasia type I	Multiple facial angiofibromas + collagenomas + *café-au-lait* spots + gingival papules + lipomas	Parathyroid adenoma + pituitary tumors + pancreatic neoplasms
Multiple endocrine neoplasia type II	Lichen amyloidosus	Parathyroid adenoma + pheochromocytoma + medullary thyroid carcinoma
Muir-Torre syndrome	Sebaceous tumors (adenomas, adenocarcinomas, epitheliomas)	Gastrointestinal + endometrial + ovarian + urothelial + biliary cancer
Peutz-Jeghers syndrome	Pigmented mucocutaneous macules + gynecomastia	Intestinal + breast + pancreatic + ovarian + testicular + cervical malignancies
von Recklinghausen's disease	*Café-au-lait* spots + axillary and inguinal freckles + cutaneous neurofibromas + plexiform neuromas	Malignant degeneration of neurofibroma + astrocytoma + glioblastoma + meningioma + bilateral pheochromocytomas

## Pruritus

It is defined as an uncomfortable feeling that triggers the urge to scratch.^40^ It can be triggered directly on the skin by mechanical or thermal stimuli or indirectly by chemical mediators or, in the peripheral nervous system, by changes in local innervation, or in the central nervous system without peripheral stimuli. The main mediators involved are histamine, neuropeptides, prostaglandins, serotonin, acetylcholine or bradykinin, and many others are being studied, such as vanilloid, opioid, and cannabinoid receptors.

It can be acute (up to 6 weeks) or chronic (longer than 6 weeks in duration). The acute type is more related to urticaria, adverse drug events and infections, whereas the chronic type is more often associated with cutaneous and systemic diseases. Chronic pruritus is more refractory to treatment, and when perpetrated in the pruritus-scratch cycle, it triggers secondary skin lesions (excoriations, pigmentation changes, lichenification) which, in the long term, interfere with sleep, emotional status, sexual desire, negatively impacting the quality of life.^41^

The investigation of pruritus begins with a detailed anamnesis. It is important to characterize the pruritus (generalized × localized, date of onset, variation throughout the day, and factors that intensify or alleviate it) and ask about drug use, recent travel, whether it affects other family members, workplace, contact with animals and /or allergens and systemic signs such as fever, night sweats, weight loss, abdominal pain, dizziness, arthritis, paresthesia. Then, the physical examination is carried out, including the genital, interdigital, eye and nipple regions, in an attempt to identify primary inflammatory or infectious lesions that can allow the diagnosis of a dermatological condition.^42^ If not identified, it is necessary to evaluate the medications being used, as they may cause pruritus due to allergy, cholestasis, cutaneous xerosis, accumulation of metabolites in the skin or nerves, and phototoxicity. If one or more of the medications are suspected, they should be discontinued for 4–6 weeks. If there is a relief, the causal link is reinforced and the drug should be changed.^43^ If there is no suspected medication, the possibility of neurological or systemic disease should be considered and laboratory tests should be requested: complete blood count; kidney, liver and thyroid functions; iron and ferritin levels; CRP and ESR. If necessary, the investigation is complemented with anti-HIV serology, tests for hepatitis B and C, chest and abdomen imaging tests, protein immunoelectrophoresis, parathyroid hormone, cholesterol and fractions, and skin biopsy.

Neurological causes include neuropathies (peripheral and central) and psychogenic causes. Peripheral neuropathies should be classified as localized (mononeuropathy) – postherpetic neuralgia (secondary lesions in the affected dermatome); notalgia paresthetica (dorsal hyperpigmented plaque, at the level of the T2–T6 vertebrae); brachioradial pruritus (pruritus and excoriation located on the sides of the arms and shoulders), and generalized (polyneuropathy): diabetic neuropathy, nutritional deficiencies, alcoholism, and autoimmune diseases. The rarer central neuropathies are due to central nervous system tumors, multiple sclerosis (xerosis, secondary lesions, with ataxia, diplopia, dysesthesia), and strokes (secondary lesions, with sensory loss, weakness, ataxia). Neuropathic pruritus can be treated topically with capsaicin and menthol-based creams and orally with gabapentin, pregabalin, mirtazapine, and paroxetine. The psychogenic types, on the other hand, are secondary to diseases such as depression, obsessive-compulsive disorder, fibromyalgia, and parasitic delusion and should be referred for psychiatric evaluation. Psychogenic causes can be considered when there is no identified somatic or cutaneous cause, the condition has lasted more than 6 months, there is a temporal relationship with psychological events, shows intensity variation with stress, predominance during rest, and relief with psychotropic drugs or therapy.^44^

The systemic causes include a myriad of diseases, with their peculiarities. Here are some examples:

1 Hepatic: primary biliary cirrhosis, hepatitis C, drug-induced cholestasis of pregnancy. Acral onset (feet and hands), with subsequent generalization. Associated signs: jaundice, telangiectasias, peripheral edema. Treatment with cholestyramine, ursodeoxycholic acid and naltrexone.
2 Renal (dialytic patients, in particular): very intense, worsens at night, may progress to nodular prurigo, Kyrle’s disease. The most affected area is the back (butterfly sign – the central area is spared), followed by the arms, head and abdomen. Treatment includes UVB phototherapy, thalidomide, and topical tacrolimus.
3 Hematopoietic: lymphoma (may be the first symptom of the disease), mastocytosis, polycythemia vera (occurs after bathing; associated signs: ecchymosis, mucosal bleeding, headache, hepatosplenomegaly), iron deficiency anemia (angular cheilitis, mucocutaneous pallor; regresses with iron replacement therapy), leukemias, multiple myeloma (ecchymosis, neuropathy, hepatosplenomegaly), myelodysplasias. Cutaneous T-cell lymphoma and Sézary syndrome cause pruritus that is difficult to control.
4 Solid neoplasms: pruritus as a paraneoplastic manifestation. It precedes or accompanies the neoplasm.
5 Endocrinological: in hyperthyroidism, it may be the first symptom of the disease; it may include fine tremors, hyperhidrosis, pretibial myxedema. In diabetes, the pruritus is more localized, present on the trunk and scalp. In mastocytosis: refractory, with the presence of urticaria, angioedema, intolerance to heat, and pulmonary, gastrointestinal, or cardiac alterations. In hypothyroidism: less frequent and closely related to xerosis; associated signs: alopecia, periorbital edema, macroglossia, weight gain, fatigue.
6 Connective tissue diseases: the medications used should be evaluated, as they can directly or indirectly cause pruritus. In dermatomyositis, Sjögren’s syndrome and lupus, the pruritus can precede the disease by up to 10 years. In scleroderma, pruritus is a late manifestation. Dermatomyositis: an important symptom of the disease; the use of photoprotection should be evaluated, as well as the side effects of medications, such as the antimalarial drug that causes reactions in up to 30% of cases. Scleroderma: the pruritus occurs more often at night and in affected areas, being aggravated by stress, fatigue, sleep, hot water and surroundings, with associated numbness and paresthesia. Treatment includes optimizing skin hydration and evaluation of the medications used (calcium channel blockers cause pruritus and can be replaced by serotonin-reuptake inhibitors), and investigating neoplasms. Sjögren's syndrome: dry eye may be pruriginous, and cutaneous xerosis aggravates the symptoms. If there is no control regarding cutaneous hydration, one should look for complications of the disease, such as vasculitis, neuropathy, primary biliary cirrhosis, autoimmune hepatitis. Lupus erythematosus: pruritus may occur even in the absence of lesions. Photoprotection should be assessed, and treatment should be optimized (smoking cessation), adverse effects should be assessed (thalidomide causes neuropathy, antimalarial drugs cause pruritus), as well as complications (nephritis, primary biliary cirrhosis, autoimmune hepatitis, and sclerosing cholangitis).^45^
7 HIV: pruritus may be associated with dermatoses that are more prevalent or aggravated by HIV infection or occur as a primary symptom of the infection, such as eosinophilic folliculitis, exacerbated reaction to insect bites, and pruritic HIV papules.

Pruritus is a common and uncomfortable symptom. Its diagnosis can be easy and objective, or it can be challenging. Its study is an ongoing process and new drugs are being developed.

## Cutaneous manifestations associated with COVID-19

Skin involvement seems to be an indirect and non-specific effect of COVID-19, as with other viral infections, regardless of the stage or severity of the disease. However, there are studies trying to prove the presence of the virus directly on the skin, resulting in cutaneous lesion. The latter would be classified as COVID-19-specific lesions.

A consistent number of case reports and clinical series from different regions of the world have been published describing a spectrum of cutaneous manifestations associated with SARS-CoV-2 infection. However, images and histopathological findings of these lesions were rarely included, making the causal correlation between the dermatological clinical manifestation and COVID-19 difficult. There is no consensus; however, the Italian group led by Paolo Gisondi suggested that cutaneous manifestations be classified into four categories.^46^

### Categories of cutaneous manifestations of COVID-19

1. Exanthema (varicella-like, papulovesicular and morbilliform skin eruption)

A study conducted by Galván-Casas et al. in Spain showed that vesicular eruptions usually appear early in the course of the disease, even before other symptoms. The chilblain-like pattern tends to appear late, while the other manifestations usually accompany other signs and symptoms of the infection.^47^

The vesicular eruption described in COVID-19 usually manifests in the early stage of the disease and is clinically characterized by monomorphic papulovesicular lesions (differentiating it from varicella), usually generalized, predominantly affecting the trunk, with or without pruritus.^48^ The lesions appear on average three days after symptoms onset, lasting approximately eight days. They are related to disease with moderate severity and usually affect middle-aged patients.^46^

The maculopapular or morbilliform exanthema is clinically similar to other viral exanthemas. It may have a perifollicular distribution, affecting axillary folds, cubital fossa, or can occasionally be disseminated. The lesions usually accompany the other viral infection symptoms and last from three to ten days. Pruritus is described in most patients and is usually associated with more severe disease and older patients.^46^, ^47^

2. Vascular manifestation (chilblain-like, petechial/purpuric and livedoid lesions)

Several types of vascular lesions have been described in SARS-CoV-2 infection, including chilblain-like, livedoid, petechial, purpuric, and necrotic lesions. Most seem to have the formation of intravascular thrombi in their pathophysiology. Cases of immune thrombocytopenic purpura (ITP) and antiphospholipid antibody syndrome (APS) have been described as cutaneous manifestations associated with COVID-19. The spectrum of vascular lesions may have different mechanisms of action, including direct action of the virus on endothelial cells and/or an indirect effect triggering immune or autoimmune reactions (as in the case of ITP). Whatever the mechanism, the resulting microvascular dysfunction can lead to increased vasoconstriction, inflammation, and a pro-thrombotic state.^46^

Chilblain-like lesions typically occur in children and adolescents and primarily affect the acral regions. They clinically manifest as erythematous-violaceous macules, which may develop into vesicles, pustules, or bullae.^49^ They are usually asymmetrical and most are accompanied by pain and/or pruritus. They affect mainly young patients in the absence of systemic symptoms. Lesions usually appear at the later stage of infection and seem to resolve spontaneously after two to four weeks.^46^ Late onset of lesions may explain possible negative viral detection results.^47^

A wide spectrum of purpuric and petechial lesions has been described as a possible association with SARS-CoV-2 infection. Lesions may appear at any time during the course of the disease and with no predominance of location. They may manifest only as petechiae or appear as erythematous-purpuric macules that coalesce to form plaques, retiform purpura and even necrotic lesions. Necrotic lesions can appear at any time during the course of the disease and are preferentially located in the lower limbs and in older patients or in the severe form of the disease. There are reports of lesions similar to livedo reticularis and acrocyanosis at varying degrees, and according to the authors, the lesions may be explained by the underlying coagulation disorders associated with COVID19.^46^, ^47^

Cases of children with signs and symptoms consistent with Kawasaki disease and laboratory evidence of recent SARS-CoV-2 infection have been reported. Some cases were provisionally termed as ‘Pediatric Multisystem Inflammatory Syndrome Associated with Sars-Cov-2 Infection’s. Such an association may corroborate the occurrence of endothelial damage associated with the viral infection.^46^, ^50^

3. Acral papular eruption

The acral papular eruption is the appearance of confluent erythematous/yellowish pruritic papules that, after a few days, form pruritic, symmetric plaques that are refractory to treatment with topical steroids. Lesions appeared about 13 days after a SARS-CoV-2 positive test and showed spontaneous resolution.^51^

4. Urticarial eruption

The urticarial pattern has been described as a mildly pruritic erythematous eruption that shows spontaneous resolution within a few days and often manifests concurrently with the other symptoms of COVID-19.^47^

Activation of mast cells and basophils, by direct or indirect viral effect, is a possible event and, therefore, cutaneous manifestations such as urticaria and exacerbation of pre-existing conditions such as atopic dermatitis may be the initial clinical signs of COVID-19. The direct cytopathic effect of SARS-CoV-2 can occur in vesicular or papulo-vesicular lesions, which are very similar to those caused by the Herpesviridae family.

In conclusion, the cutaneous manifestations associated with SARS-CoV-2 infection can be multiple and have a specific etiology, can be difficult to prove as far as etiology or, more often, can be paraviral, due to the inflammatory phenomena released in the course of the infection, or even due to exposure to drugs during the prodromal period or during the treatment of the disease.^52^

## Financial support

None declared.

## Authors’ contributions

Juliana Martins Leal: Drafting and editing of the manuscript; approval of the final version of the manuscript.

Gabriela Higino de Souza: Drafting and editing of the manuscript; approval of the final version of the manuscript.

Paula Figueiredo de Marsillac: Drafting and editing of the manuscript; approval of the final version of the manuscript.

Alexandre Carlos Gripp: Drafting and editing of the manuscript; approval of the final version of the manuscript.

## Conflicts of interest

None declared.
